# A Longitudinal Multimodal Imaging Study in Patients with Temporo-Insular Diffuse Low-Grade Tumors: How the Inferior Fronto-Occipital Fasciculus Provides Information on Cognitive Outcomes

**DOI:** 10.3390/curroncol31120595

**Published:** 2024-12-20

**Authors:** Barbara Tomasino, Cinzia Baiano, Giuseppe Kenneth Ricciardi, Marta Maieron, Andrea Romano, Ilaria Guarracino, Miriam Isola, Maria De Martino, Serena D’Agostini, Daniele Bagatto, Teresa Somma, Miran Skrap, Tamara Ius

**Affiliations:** 1 Scientific Institute, IRCCS E. Medea, Dipartimento/Unità Operativa Pasian di Prato, 33037 Pasian di Prato, Italy; barbara.tomasino@lanostrafamiglia.it (B.T.); ilaria.guarracino@lanostrafamiglia.it (I.G.); 2 Division of Neurosurgery, Department of Neurosciences, Reproductive and Odontostomatological Sciences, Università degli Studi di Napoli Federico II, 80138 Napoli, Italy; cinzia.baiano@unina.it (C.B.); teresa.somma@unina.it (T.S.); 3 Neuroradiology Departments, Azienda Ospedaliera Universitaria Integrata Verona, Ospedale Civile Maggiore, Borgo Trento, 37126 Verona, Italy; 4 Department of Physics, University Hospital of Udine, 33100 Udine, Italy; marta.maieron@asufc.sanita.fvg.it; 5 NESMOS Department, U.O.C. Neuroradiology “Sant’Andrea” University Hospital, 00189 Rome, Italy; andrea.romano@uniroma1.it; 6 Division of Medical Statistic, Department of Medicine (DAME), University of Udine, 33100 Udine, Italy; miriam.isola@uniud.it (M.I.); maria.demartino@uniud.it (M.D.M.); 7 Neuroradiology Unit, Department of Diagnostic Imaging, University Hospital of Udine, 33100 Udine, Italy; serena.dagostini@asufc.sanita.fvg.it (S.D.); daniele.bagatto@asufc.sanita.fvg.it (D.B.); 8 Neurosurgery Unit, Head-Neck and NeuroSciences Department University Hospital of Udine, 33100 Udine, Italy; skrap@asufc.sanita.fvg.it

**Keywords:** predictive value, lower-grade gliomas, inferior fronto-occipital fasciculus, microstructure analysis, fiber tracking, neuropsychology

## Abstract

Background: Tractography allows the in vivo study of subcortical white matter, and it is a potential tool for providing predictive indices on post-operative outcomes. We aim at establishing whether there is a relation between cognitive outcome and the status of the inferior fronto-occipital fasciculus’s (IFOF’s) microstructure. Methods: The longitudinal neuropsychological data of thirty young (median age: 35 years) patients operated on for DLGG in the left temporo-insular cortex along with pre-surgery tractography data were processed. Results: A degraded integrity of the left (vs. right) IFOF (lower fractional anisotropy and length, *p* < 0.001; higher mean and axial diffusivity, *p* < 0.01) was found, with lower microstructural variables in the infiltration (vs. dislocation) group. Significant decreases immediately post-surgery vs. pre-surgery mainly occurred in lexico-semantics (*p* < 0.001), with significant improvements at follow-up in all the tests (*p* < 0.01 to *p* < 0.001), despite values in the range of 44% to 47.82% of patients with below cut-off scores regarding naming verbs and making visual lexical decisions. The status of left and right IFOFs is predictive of a decrease in immediate post-surgery performance for several tests (*p* < 0.05); similarly, it is predictive of better recovery in the follow-up performance for naming nouns, naming verbs, making phonological fluency lexical decisions, and the token test (*p* < −0.05). For the ROC analysis, a significant result was obtained for the verb-naming test, with a cut-off of 79%. Conclusions: This study supports the role of the predictive value of pre-operative tractography for assessing the immediate post-operative result and at follow-up the risk of developing a cognitive deficit.

## 1. Introduction

White matter (WM) alteration emerges as a crucial factor in patients with low-grade gliomas (LGGs). LGGs, although considered less malignant, intricately infiltrate the surrounding WM tracts, disrupting the neural connectivity essential for various cognitive and motor functions. The insular cortex is one example of such an intricate area. Due to its location, the insular cortex is highly connected with surrounding cortical areas. Its resection has traditionally been viewed as hazardous [1]. Indeed, the major difficulty of performing a gross total resection is represented by the tendency of insular LGGs to spread along the intricate network of afferent and efferent connections [2,3]. The inferior fronto-occipital fascicle (IFOF) runs inferiorly to this area [4]. It is one of the longest association fiber tracts of the brain, representing the ventral pathway of the dual-stream model of language processing, and it is often found altered in LGGs involving the insular cortex.

The pre-operative evaluation of the anatomical relationships between the lesion and WM tracts is thus critical to plan surgical entry, the extent of resection, and to minimize the risk of neurologic impairments [5,6]. Post-processing analyses provide both qualitative and quantitative information relating to the subcortical WM. Qualitative information is useful for surgical planning to detect the trajectories of fascicles, their position with respect to the tumor, and any interruptions or changes in direction [7]. Quantitative analysis, based on the calculation of different microstructural variables, is useful for comparing healthy and pathological anatomies through predictive indices [8]. In this clinical setting, tractography is a potential tool for providing pre-surgical predictive indices able to discriminate among patients with different post-operative outcomes [9,10]. The classically used variables to probe the microarchitecture of the WM in different pathological states are fractional anisotropy (FA); the number of streamlines (NS); and the radial, axial, and mean diffusivity measures (RD, AD, and MD, respectively). FA and NS are the first measures used in the study of WM and they are considered indexes of directionality and structural integrity [11,12]. In recent years, studies on fiber-tracking methods have also analyzed the aforementioned scalar diffusivity measures of which the MD is a synthesis [13].

The purpose of this study was to explore longitudinally the clinical value of an integrative tractography approach in patients with temporo-insular diffuse low-grade gliomas (DLGGs) in the left hemisphere. We evaluated longitudinally their pre-operative, immediately post-operative, and follow-up cognitive status and the relation to the integrity of the IFOF. We then assessed the role of tractography as a predictive tool. Understanding the specific WM changes would allow for more nuanced surgical planning, rehabilitation strategies, and prognostic assessments.

## 2. Materials and Methods

### 2.1. Study Population

We retrospectively reviewed a consecutive series of patients surgically treated for DLGGs between 2012 and 2018. Thirty cases met the following strict inclusion criteria:

- DLGG in the temporo-insular left dominant hemisphere;
- Age ≥ 18 years;
- No previous surgery;
- No pre-operative chemo- or radiotherapy;
- Availability of pre-operative 3-Tesla MRI, including DTI;
- Revision of histopathological specimens by using the 2021 [14] WHO Classification of Tumors of the Central Nervous System;
- Intra-operative multimodal surgical protocol based on awake surgery, brain mapping, neurophysiological monitoring, and intra-operative real-time neuropsychological testing;
- Pre- and post-operative neuropsychological evaluation at one week and four months of follow-up;
- Needle biopsies were excluded from the study.

This study was approved by the Single Regional Ethics Committee of Friuli-Venezia Giulia, with the protocol number: 0036567/P/GEN/EGAS, study ID 2540. Considering the retrospective nature of the study, written consent to participate in the study was not applicable. Written informed consent was obtained for surgery.

### 2.2. MRI Data Acquisition

A 3T MR system (Achieva, Philips medical system) and a SENSE eight-element phased array head coil were used to acquire anatomical and DTI images, which lasted about 20 min. The details for acquisitions of T2-weighted images are: TR/TE = 2500/368.328 ms; FOV = 240 mm; 190 sagittal slices; voxel size, 1 × 1 × 1 mm, while for acquisition of post-gadolinium contrast T1-weighted images they are: TR/TE = 8.100/3.707 ms; FOV = 240 mm; 190 sagittal slices; voxel size = 1 × 1 × 1 mm. Acquisition was optimized for the standard pre-operative clinical protocol adopted by the Department of Neuroradiology of the Hospital Santa Maria della Misericordia of Udine. Single-shot echo-planar DTI sequence details are: whole brain coverage, TR/TE = 8.800/74 ms; FOV = 224 mm; 54 contiguous axial slices; voxel size; 1.8 × 1.8 × 2.2 mm; b0 and b1000 s/mm^2^; 64 non-coplanar images. The gradient directions were uniformly distributed on a sphere.

### 2.3. MRI Structural Data Analyses

We drew lesion volumes of interest (VOIs) on the patients’ T2 MRI scans by using MRIcron software (v. 1.0.20190902, https://www.nitrc.org/projects/mricron). We used the “Clinical Toolbox” (https://www.nitrc.org/projects/clinicaltbx/) for SPM12 (https://www.fil.ion.ucl.ac.uk/spm/) (all accessed on 10 December 2024) to normalize the patients’ VOIs to the Montreal Neurological Institute (MNI) space. To rule out the possibility that the normalization process was incorrect, a visual examination of the output was conducted. After that, we used the MRI-cron procedure to overlap the VOIs in order to create a maximum lesion overlay map. The process produces a percentage overlay plot that displays the results on a color scale.

### 2.4. Fiber-Tracking Method

Raw diffusion images were corrected for eddy current distortion using the FSL-6.0.7 version software library and processed with the MATLAB platform-based software Star Track (Spherical Deconvolution Diffusion Tractography tools) [15] (https://www.mr-startrack.com/, accessed on 10 December 2024) using a damped Richardson–Lucy algorithm (Dell’acqua 2010) and applying the following SD algorithm parameters: iterations = 350, n = 0.06, r = 8. We generated a spherical deconvolution deterministic tractography used to obtain local tensor orientation estimates and fractional anisotropy maps and to perform fiber tracking. Deterministic whole-brain fiber tracking was performed in each subject’s native space using the Fiber-Assignment by Continuous Tracking algorithm [16]. TrackVis software (ver. 0.6.1, http://trackvis.org/, accessed on 10 December 2024) [17] was applied to perform deterministic tractography of the white matter pathways for SD models, to perform virtual dissection using path filter tools, and to select groups of trajectories and simultaneously display flow lines and FA maps (FA value of 0.15, minimum fiber length of 200 mm, and maximum angle of 45°). In the first step, an initial selection of the IFOF streamlines was performed drawing a single region of interest through the anterior part of the external capsule on a coronal cut at the level of the anterior commissure. The criteria for including flow lines within the ROI were judged concerning: (1) their location and proximity to the core of the fascicle and (2) the cohesive shape of the flow lines to those within the bundle core. The approach was purely anatomical and based on the FA color map. Trajectories that could not belong to IFOF in terms of size, angle, and path were then excluded with “not” regions of interest. “Not” regions were introduced in TrackVis to eliminate features judged to be external to the IFOF by experienced radiologists.

### 2.5. Quantitative and Qualitative Analysis

**Figure 1:**
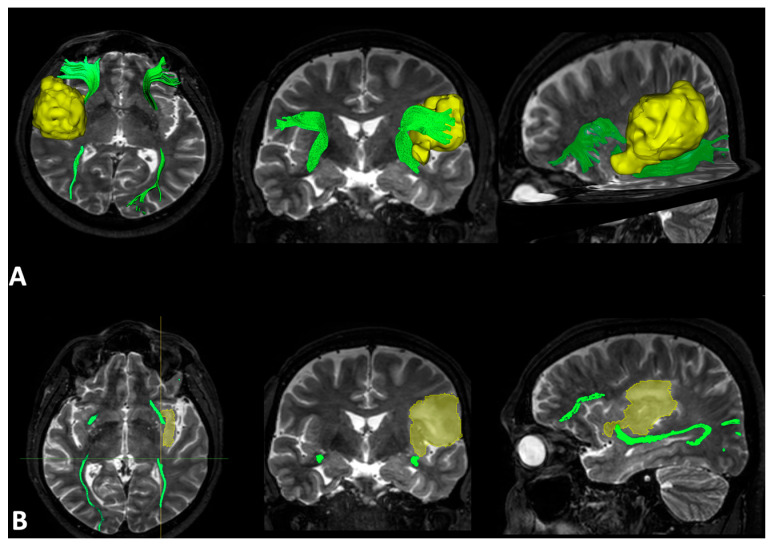
Left hemisphere temporo-insular low-grade glioma: (**A**) the qualitative analysis shows left IFOF (in green) downward displacement in axial and sagittal planes in 3D neuroradiological view of the tumor (in yellow). (**B**) T2-weighted sequences showing axial, coronal, and sagittal view of the tumor (in yellow) and the course of IFOF in the healthy and tumoral hemisphere (in green).

After IFOF extraction, the quantitative analysis evaluated the number of streamlines (NS), the number of voxels (NV), the fascicle volume (V), the length (L), the average angle (A), the fractional anisotropy (FA), the axial diffusivity (AD), the mean diffusivity (MD), and the radial diffusivity (RD). The qualitative analysis identified two groups of patients: those for which the fascicle was traceable and displaced by the tumor, and those for which there was a partial absence of trajectories because of tumor infiltration (see Figure 1).

### 2.6. Neuropsychological Evaluation

All patients received a neuropsychological evaluation by a standardized protocol the same day as the MRI. Post-operative evaluations were carried out one week and four months after surgery. Different cognitive domains were evaluated as follows. Fluid intelligence was measured by Raven matrices [18]; executive functions were tested by the backward version of the digit span test, as a measure of working memory [19], and by verbal fluency [20]; praxis was tested by two tasks of imitation for arm/hand movements (ideomotor apraxia) and orofacial (oral praxis) [21,22]; short-term memory was assessed using a digit span task [19]; language was tested using the battery for the assessment of aphasic disorders. In particular, we used: reading/writing/repetition (of words and pseudo-words) [23], lexico-semantics (using object naming [23], verb naming [23], pyramids and palm trees test [24], visual and auditory lexical decisions [23]), and comprehension (phonological discrimination [23], auditory understanding of objects and verbs [23], verbal comprehension using of the token test [25]).

### 2.7. Intra-Operative Surgical Protocol

The surgical procedures were performed using cortical and subcortical mapping methods by the usual surgical technique. In addition to direct electrical stimulation, a performance-based resection was performed by using real-time neuropsychological testing [26]. Surgical resection was stopped in case of positive direct electrical stimulation at both cortical and subcortical levels or in case of an important decrease in real-time neuropsychological testing scores. The integration with real-time neuropsychological testing allows bridging the information gap between two contiguous direct electrical stimulations.

### 2.8. Histological and Molecular Analysis

All histological specimens were reviewed and classified by the 2021 [14] WHO brain tumor classification.

### 2.9. Statistical Analyses

The categorical variables were described through absolute frequencies and relative percentages. Continuous variables were synthesized by reporting mean and standard deviation, or median and IQR, depending on the type of distribution of the variable. The Gaussian distribution was analyzed using the Shapiro–Wilk test. For the comparisons of the quantitative variables of tractography between healthy and pathological hemispheres and between infiltrated and displaced fascicles, the Student’s *t*-test or the Mann–Whitney U test was used, depending on the distribution of the variables. In the case of multiple comparisons, Bonferroni correction was applied. Pearson’s correlation index or Spearman’s correlation coefficient was used to evaluate the association between the quantitative variables of tractography and neuropsychological test scores. Linear regression was also used to evaluate the association between MD and percentage accuracy of performance of all neuropsychological test scores. The ROC analysis was used to identify the percentage of NS associated with a lower performance accuracy in neuropsychological post-surgical tests (at 1 week post-surgery and 4-month follow-up). The inter-hemispheric difference in NS (idNS) = 1 − (NS left/NS right) variable was therefore defined. The variable idNS = 1 − (NS left/NS right) was compared with the dichotomous scores (i.e., pathological vs. normal score) and with percentage accuracy of performance of all neuropsychological test scores through a ROC analysis. This variable indicates the percentage of untraceable NS in the left hemisphere compared to the normal right side and it assumes values between 0 and 1. Median (idNS) = 0.64; interquartile range (IQR) (idNS) = (0.34–0.94); average (idNS) = 0.59; standard deviation (SD) (idNS) = 0.36. The significance of all tests was evaluated considering a *p*-value < 0.05 as a cut-off. Analyses were performed using Stata/IC 17.0 (Stata Corp LP, College Station, TX, USA).

## 3. Results

### 3.1. Clinical Results

The demographic and pre-operative clinical characteristics of the study population are summarized in Table 1. In all cases, preoperative MRI showed temporo-insular lesions with hyperintensity on T2-weighted images and hypointensity on T1-weighted images without any site of contrast enhancement after gadolinium injection. All lesions were associated with infiltrative patterns inside the white matter without significant mass effect.

### 3.2. MRI Structural Data Analysis

**Figure 2:**
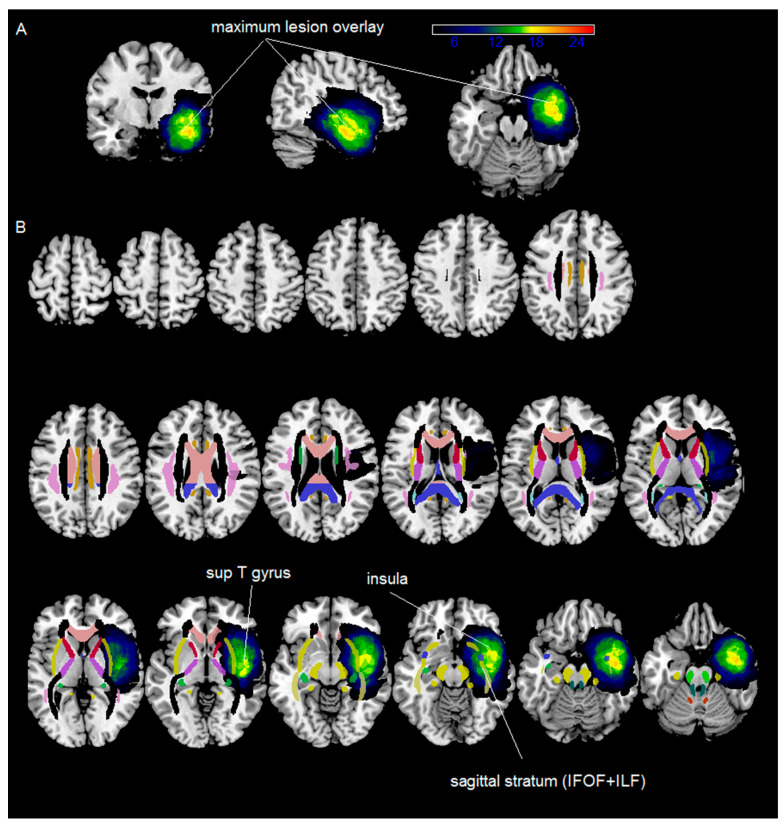
Brain areas that were maximally invaded by the tumor in the whole patient sample. (**A**) shows the maximum lesion overlay of all the patients’ volumes of interest drawn on their lesion volume. The color bar represents the number of cases, with yellow–orange indicating the maximum overlay. (**B**) shows the same maximum lesion overlay which is displayed on axial slices of a T1-weighted template, along with the Johns Hopkins University (JHU) Diffusion Tensor Imaging (DTI)-based white matter atlases [27]. The colors represent the different white matter pathways according to the atlas. Data are displayed in neurological conventions.

The maximum lesion overlay occurred with a 70% overlap among the group in the left insula, part of the superior/middle temporal gyrus, the amygdala, and the temporal pole (see Figure 2 and Table 2). At the subcortical level, the maximum lesion overlap occurred on part of the left sagittal stratum (IFOF + ILF) (with a 65% overlap across the patients).

### 3.3. Neuropsychological Data

Pre-surgery, patients’ performance was within the normal range for almost all the tests, except that for some tasks a percentage of patients fell below the normal range (i.e., working memory, naming nouns, naming verbs, auditory lexical decisions, and visual lexical decisions, see Table 3).

The Friedman test determined whether patients’ accuracy differs before, immediately post-surgery, and at follow-up. Post hoc pairwise comparisons (see Table 4) showed that there were transient changes in the patient’s level of performance. Indeed, follow-up vs. pre-surgery did not differ significantly. Significant decreases at 1 w vs. pre-surgery were found in the executive function domain (short-term memory), semantics (pyramids and palm trees test, noun naming, verb naming), writing, comprehension (token test), and praxis (ideomotor apraxia).

Their level of performance immediately post-surgery showed impairments in the lexico-semantic processing domain for which we detected the higher percentage of patients with below cut-off scores (above 40% of cases in noun naming, verb naming, fluency, visual and auditory lexical decisions, see Table 3).

**Figure 3:**
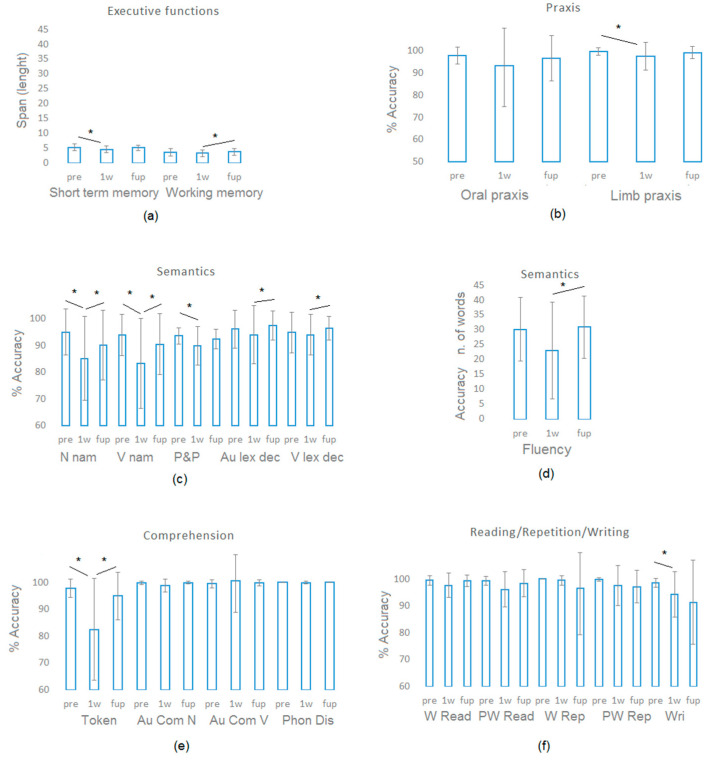
Patients’ performance before (pre), immediately post-surgery (1 week after, 1 w), and at follow-up (fup) assessment. (**a**) For executive functions the length of the short-term memory and working memory span is displayed; (**b**) Oral and limb (ideomotor apraxia), % of correct responses is shown; (**c**) % of correct responses in tests tapping semantics (N nam = noun naming; v nam = verb naming; P&P = Pyramids and Palm Trees Test; Au lex dec and V lex dec = auditory and visual lexical decisions); (**d**) for verbal fluency the number of words retrieved is displayed; (**e**) % of correct responses in tests tapping comprehension (Token = Token test; Au Com N and Au Com V = Auditory comprehension of nouns and verbs; Pho Dis = phonologic discrimination); (**f**) % of correct responses in tests tapping reading, repetition, and writing (W = words; PW = pseudowords; Read = reading; Rep = repetition; Wri = writing). Asterisks * denote significant changes.

At follow-up, despite an improvement in the level of performance found in working memory, fluency, naming verbs and nouns, token test, and auditory and visual lexical decisions (see Table 4 and Figure 3), the level of performance showed impairment in the lexico-semantic processing domain for which we detected the higher percentage of patients with below cut-off scores (naming verbs, 44% of the patients; visual lexical decisions, 47.82% of the patients).

### 3.4. DTI Quantitative and Qualitative Analysis

The microstructural analyses showed a degraded integrity of the left IFOF present pre-surgery (see Table 5). We found that all the microstructure variables of the IFOF significantly differed according to the hemisphere. FA was significantly lower in the tumor hemisphere (0.42 vs. 0.47, *p* < 0.001), as well as L (126 vs. 140, *p* < 0.001). By contrast, MD (0.83 vs. 0.78, *p* < 0.001), AD (1.31 vs. 1.25, *p* < 0.01), and A (3.79 vs. 3.38, *p* < 0.05) were significantly higher in the tumor hemisphere. In the tumor hemisphere, FA was always inversely proportional to the diffusivity measures: AD (r = −0.438, *p* < 0.022), MD (r = −0.657, *p* < 0.076), RD (r = −0.771, *p* < 0.001). NS, NV, V, and LS (all *p* < 0.001) as well as the FA (*p* < 0.01) were significantly lower in the infiltration group compared to the dislocation group. By contrast, the RD was significantly higher in the infiltration group compared to the dislocation group (*p* < 0.05).

### 3.5. Association Between Neuropsychological Altered Performance and Microstructure Analysis

#### 3.5.1. Immediate Post-Surgery Data

We calculated the delta pre-surgery–immediately post-surgery for those tasks showing a deflection at immediate post-surgery assessment. We found that the status of the IFOF is predictive of a decrease in immediate post-surgery performance for naming verbs, token test, naming nouns, pyramids and palm trees test, short-term memory, writing, and ideomotor apraxia (See Table 6).

#### 3.5.2. Follow-Up Data

**Figure 4:**
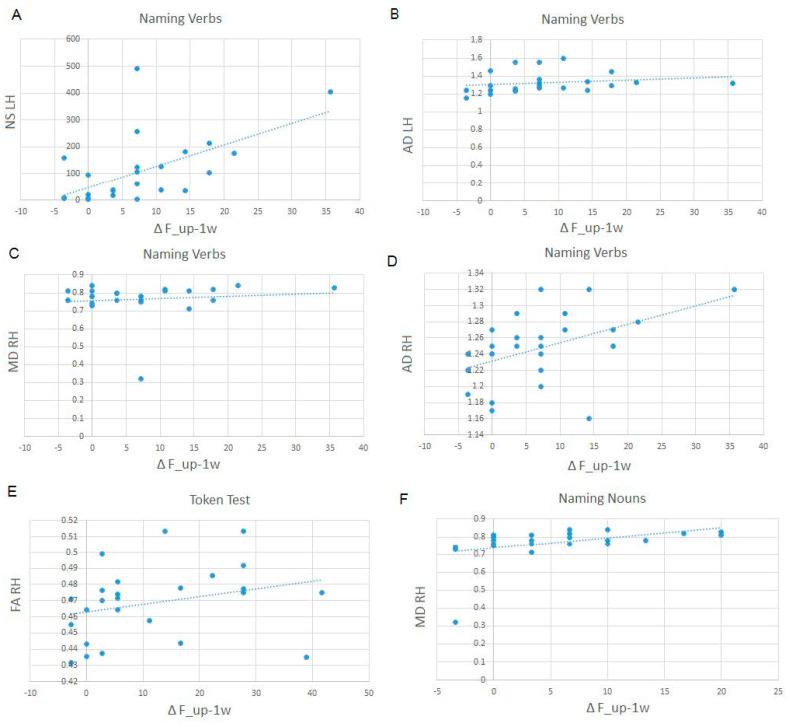
Examples of association between IFOF status and performance at follow-up for naming verbs (**A**) shows the relation to NS of the LH, (**B**) shows the relation to AD of the LH, (**C**) shows the relation to MD of the RH and (**D**) shows the relation to MD of the RH), Token Test (**E**) shows the relation to FA of the RH, and Naming nouns (**F**) shows the relation to MD of the RH.

We calculated the delta follow-up–immediately post-surgery for those tasks showing a deflection at immediate post-surgery assessment. We found that the status of the IFOF is predictive of a better recovery in follow-up performance for naming nouns, naming verbs, phonological fluency, lexical decisions, and the token test (see Table 6 and Figure 4).

#### 3.5.3. Predictive Analyses Based on Number of Streamlines on the Immediately Post-Surgical Cognitive Data

**Figure 5:**
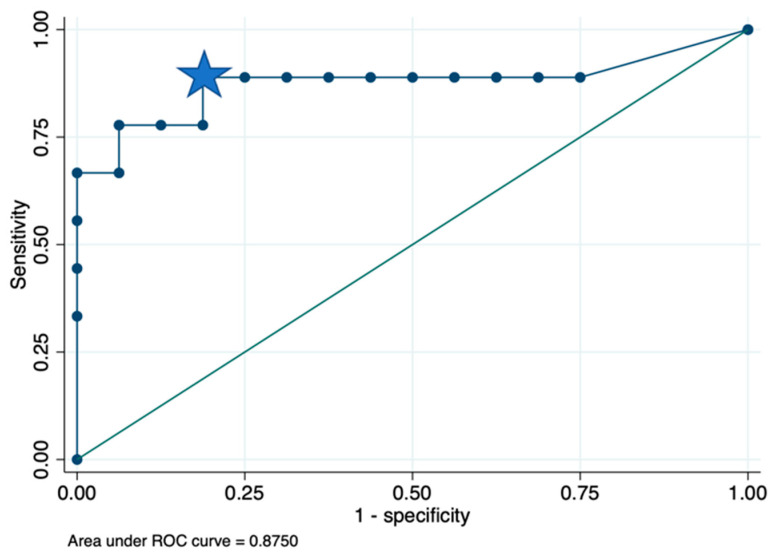
Receiver operating characteristic (ROC) analysis showed that the area under the curve of the verb naming at the immediate post-surgery evaluation was 0.8750 (CI 95% 0.68326–1). Predictive accuracy was 84% naming immediately post-surgery. (It is represented by the closest point to the top left corner of the graph, indicated by the blue star).

The optimal cut-off value was found for verb naming at the immediate post-surgery evaluation, which corresponded to 0.79 (see Figure 5). It was the point with the highest sensitivity (88.9%) and specificity (81.2%), with a resulting area under the curve of 0.8750 (CI 95% 0.68326–1) and a predictive accuracy of 84%. This result indicates that a reduction of more than 79% of left IFOF streamlines is associated with a higher probability of developing a pathological score at the immediate post-surgery evaluation.

Furthermore, patients with a reduction of more than 79% of left IFOF streamlines have also a higher median value for the verb-naming test at follow-up compared to the others (100 IQR (92.8–100) vs. 89.3 IQR (85.7–92.9), *p* = 0.001).

## 4. Discussion

Considering the young age and long life expectancy of DLGG patients, it is mandatory to develop new diagnostic strategies both for surgical planning and pre-operative estimation of surgical risks [9,28]. Recent investigations have widely demonstrated that a multimodal surgical approach can provide a safer and wider removal of DLGG [6]. This surgical approach based on awake surgery in combination with direct electrical stimulation, real-time neuropsychological testing, transcranial magnetic stimulation, and functional MRI/tractography images fused in a neuronavigation system allows continuous intra-operative functional feedback, thus permitting maximization of the resection. In this study, quantitative data regarding pre-surgery microstructural WM changes were integrated with neuropsychological profiles to investigate WM status in patients with left cerebral hemisphere DLGG involving the temporo-insular area. Furthermore, tractography was evaluated as a predictive tool.

### 4.1. Longitudinal Cognitive Assessment

We found that DLGG involving the left temporo-insular area causes a transient decrease in the level of performance in several neuropsychological tests, mainly involving the lexico-semantic domain. The level of performance decreases at the immediate post-surgery assessment and increases at follow-up. As to the transient deficits, we would argue that the reason for observing them could be related to the effect of surgical manipulation or tissue edema; in some cases, a pre-surgery borderline performance could in principle decrease towards impairment in the immediate post-surgery assessment. Plasticity-related mechanisms could then intervene, helping functional restoration.

Nonetheless, despite this recovery, the patients’ level of performance resulted in pathology in the lexico-semantic processing domain for which we detected a higher percentage of patients with below cut-off scores (naming verbs, 44% of the patients; visual lexical decisions, 47.82% of the patients). Interestingly, in the present patient sample, performance for almost all the tasks stayed >90%; for comprehension (token test) and naming (nouns and verbs) the level at the immediate post-surgery assessment was lower compared to the other tasks (82%, 85%, and 83%, respectively). We speculate that the intra-surgical testing approach has contributed to closely monitoring the patient’s cognitive level. Taken together, the present results confirm the role of follow-up neuropsychological assessment allowing the longitudinal monitoring of patients’ cognitive status, especially when in the immediate post-surgery testing some decrements arise. It is conceivable to design even longer follow-up studies to extend patients’ monitoring. Such longitudinal assessments may clarify the long-term impact of surgery on cognitive functions.

### 4.2. Microstructural Status of the Left and Right IFOF

We a priori focused on the IFOF, as this white matter fascicle has a very high DTI tractography specificity for its identification [7]. Castellano et al. [29] found two scenarios in the pre-surgical DTI maps, either infiltration, in large fronto-insular or temporo-insular lesions, leading to a worse surgical outcome and partial resection, or an external course of the fascicle and a large removal. In our study, the structural MRI analysis showed that the maximum lesion overlay occurred in the left insula, part of the superior/middle temporal gyrus, part of the amygdala, and temporal pole. At the subcortical level, the maximum lesion overlap occurred on part of the left sagittal stratum (IFOF + ILF). Thus, our sample involved quite large insular/temporal lesions. Indeed, as predicted by Castellano et al. [29] patients showed a decrease in performance already at the pre-surgery neuropsychological assessment in some of the domains, especially in the lexico-semantic ability. The microstructural analyses showed a picture of degraded integrity of the left IFOF (as compared to the healthy hemisphere) present pre-surgery. In line with current literature [11], in our series, a reduction in trajectories obtainable with a reduction in FA corresponds to a loss of integrity and directionality of the fascicle in the tumoral hemisphere. In previous studies concerning the behavior of WM status and glial lesions, two different configurations of qualitative patterns have been described: the first in which there is a decrease in FA and NS compared to the healthy hemisphere (possible dislocation) and a second in which there is a decrease in FA associated with partial or total absence of trajectories (possible infiltration) [30,31]. In line with this, with a qualitative analysis, we were able to distinguish two groups: (1) the cases in which IFOF was simply dislocated with RD being significantly lower and (2) the cases in which there was a partial absence of trajectories, indicating a possible infiltration with significantly lower NS, NV, V, LS, and FA. To date, at the neuropsychological level, the same analysis failed to return significant differences, likely due to limited variation in the data points and to a limited sample size of the two groups.

### 4.3. DTI Tractography as a Predictive Tool

The use of tractography for predictive purposes is an open issue [12,32]. We found that some microstructural DTI variables depicting the pre-surgery status of the IFOF are predictive of the outcome for some tasks. We acknowledge that we did not measure the predictive power of baseline language performance or language symptoms at presentation, thus we cannot disentangle whether white matter measures of the IFOF were more predictive of recovery potential than behavioral variables. Future studies addressing this issue have to be planned.

We do not address the role and meaning of single parameters, as this is beyond the scope of the present work. We speculate that immediate post-operative decreases and follow-up recoveries were related to both the microstructural status of the left pathological and the right healthy IFOF.

In relation to strengthening the predictive role of the DTI data, we also found that a pre-surgery reduction of more than 79% of left IFOF streamlines correlated with a higher probability of developing a pathological score at the immediate post-surgery evaluation for verb naming, which was indeed the test for which we found a higher percentage of pathological cases in immediate post-surgery testing (64%). In the immediate post-surgery assessment patients are tested 1 week post-surgery, thus in an acute phase. Their pathological performance is often described as transient. This is also our case, as we found that patients had improved in the follow-up testing. Concerning the few post-operative reports about the language performance in patients with a glioma in the language-dominant fronto-temporo-insular area, the authors generally described mild deficits which persisted at follow-up, although with a general improvement with respect to the immediate post-operative assessment [33,34].

Lastly, as to the involvement of the right healthy hemisphere, it could be speculated that in these patients the involvement of the right hemisphere could have been determined by a high degree of infiltration of the left IFOF. We can speculate also that cognitive functions with DLGG growth centered on the left temporo-insular area are also related to a degree to the microstructural status of the right contra-lateral counterpart, which participates in maintaining, or in the case of follow-up performance, restoring, patients’ cognitive abilities. In a previous study [35], patients with insular lesions were compared to healthy controls during object naming and an involvement of the contra-lesional hemisphere in the pre-operative phase was already observed. A similar result is found in [36] who reported that patients with fronto/temporal and possibly insular tumors increased recruitment of the right hemisphere in the post-operative stage.

As to whether the deterministic or probabilistic approach is more suitable, we acknowledge that some authors have hypothesized that probabilistic tracking could lead to more accurate results because it more efficiently extracts information from the underlying data. In our study, we believe that the algorithms implemented in Startrack could better resolve the uncertainty due to the presence of fibers that may cross within each voxel, with respect to using a standard deterministic tractography approach not based on an SD analysis. Therefore, we can argue that our choice to conduct SD-based deterministic tractography could be a good representation of the anatomical path of the IFOF.

### 4.4. Clinical Value

The representation of white matter pathways measured in vivo by non-invasive MR diffusion imaging may be a promising potential predictive tool [9,10]. The integration between quantitative fiber-tracking analysis of the WM microstructure and functional outcome allows for providing a pre-surgical estimation of post-operative deficits in DLGG patients, especially for those with lesions in the dominant hemisphere [10,37]. Indeed, the pre-operative risk predictions of cognitive outcome should not simply rely on anatomical data, mainly considering the high inter-individual variance of functional language anatomy [9]. Consequently, it is of utmost importance to develop a reliable non-invasive pre-operative method to estimate the risk of post-operative deficits in the clinical management of these patients, taking into account the individual variability of connections between the cortical areas [10,38,39,40]. The estimation of the impact of surgery for infiltrating slow-growing tumors on post-surgical outcome and potential post-surgical recovery by objective measures is a cornerstone of good clinical practice [9,41]. In recent studies by Ius et al. a numerical index based on the difference between the NS of the corticospinal tract and of the IFOF trajectories of the healthy hemisphere and those of the injured hemisphere to predict the onset of post-operative neurological alterations was proposed. In these studies, predictive accuracy of the NS index was compared with more classic indices, such as MRI tumoral pattern estimates, as well as pre-operative and intra-operative evoked potentials, to confirm its value as an indicator of clinical outcome [9,10]. Another study concerning arcuate fasciculus and the predictive value of FA speculates that this could be a predictor for post-operative language recovery following tumor resection [12]. In 2021, Tuncer et al. proposed a tractography-based risk stratification model for gliomas in the language area to identify “functional nodes” in the left temporo-insular area associated with different language deficits [7]. In clinical practice, this result makes it possible to provide additional information in pre-operative counseling.

Lastly, we would like to remark that a good recovery at follow-up is likely related to the intra-operative monitoring approach. All the patients of the present series underwent awake surgery procedures accompanied by DES and real-time neuropsychological testing (RTNT) [26]. The peculiarity of RTNT is that it is performed throughout the whole resection procedure, during which a specific neuropsychological battery is continuously administered to alert the neurosurgeon to whether a decrease in performance is recorded while stimulating a given area to be resected.

### 4.5. Lexico-Semantic Processing Is Supported by the IFOF

The patients’ main changes in performance occurred in the lexico-semantic domain, consistently with the role of the IFOF in the lexical and semantic domain of the language network [42,43]. The ventral IFOF pattern is involved in the mapping of sounds based on meaning and therefore in semantics and understanding [43]. Tasks like naming nouns and naming verbs are often related to the status of the IFOF. For example, in a study on 99 patients with lesions in the left temporal–frontal–parietal cortex, it has been shown that noun- and verb-naming deficits also depended on a disconnection phenomenon: at the subcortical level, noun-naming impairments were related to damage to parts of the sagittal stratum (including the inferior fronto-occipital fasciculus and the inferior longitudinal fasciculus) among other fascicles [44]. The novelty of the present study consists of having introduced additional lexico-semantic tasks, such as lexical decisions, and having shown their significant association with the microstructure variables of the left and right IFOF. Data in the DTI literature on lexical decisions are controversial, and they have been related to the superior longitudinal fasciculus [45] and the frontal aslant tract [46] or the sagittal stratum (IFOF + ILF) [47].

### 4.6. Limits of the Study

A main limitation of this study is represented by the relatively small number of patients, which prevented us from running more in-depth analyses. Nonetheless, it is acknowledged that DLGG is rare, and this is a monocentric study.

Regarding the methodological approach, it is important to note that indices regarding WM structural changes are based on mathematical techniques that vary according to the parameters set, so it is not a direct representation of the anatomy.

Another limitation of this study is the lack of a comparison between behavioral variables (baseline language performance or language symptoms at presentation) and white matter measures of the IFOF to test which were more predictive of recovery potential. Future studies addressing this issue have to be planned.

## 5. Conclusions

This preliminary investigation sheds light on a new potential role of pre-operative quantitative tractography analysis in investigating WM microstructural changes as predictors of cognitive outcome. The structural WM changes in the tumoral hemisphere were significant predictors of the lexico-semantic processing subserved by the IFOF. The quantitative analysis may represent a promising predictive approach for patient counseling and risk assessment before surgery.

## Figures and Tables

**Table 1:** The baseline demographic and pre-operative clinical characteristics of the study population.

Parameters	Value
No. of patients	30
Sex	
Male	17 (56.6%)
Female	13 (43.3%)
Age (years)	
Median (range)	35 (20–60)
Onset symptoms	
Seizures	24 (80%)
Focal seizures	14 (46.6%)
Focal to bilateral tonic–clonic seizures	13 (43.3%)
Language symptoms	-
No symptoms (incidental LGG)	3 (10%)

**Table 2:** Anatomical localization of the maximum lesion overlays of the patient group. The number (N > 0) and the % (%N > 0) of damaged voxels for each region as reported in the brain atlas of grey (AAL) and white matter (JHU and NatBrainLab) are reported, along with the center of mass (Max X, Y, and Z MNI coordinates). Max is the number of patients with a lesion in the given area (along with the %).

Area	N > 0	%N > 0	Max	MaxX	MaxY	MaxZ	% of Patients
Insula_LH	14,128	1	19	−46	−5	−1	73.07
Temporal_Sup_LH	20,488	0.811	19	−47	−5	−1	73.07
Amygdala_LH	1965	1	18	−34	3	−20	69.23
Temporal_Mid_LH	19,609	0.555	18	−46	−1	−26	69.23
Temporal_Pole_Sup_LH	9686	0.90	18	−37	6	−24	69.23
Hippocampus_LH	7133	0.93	18	−37	−6	−20	69.23
Temporal_Inf_LH	23,508	0.82	17	−45	−5	−26	65.38
Temporal_Pole_Mid_LH	8934	0.94	17	−45	9	−22	65.38
Sagittal stratum	2231	1	17	37	−9	−19	65.38

**Table 3:** Percentage of patients with below cut-off scores in the different tasks.

		Pre	Immediately Post-Surgery	Follow-Up
Executive functions	Short-term memory	17.39	34.78	17.39
	Working memory	28.57	28.57	9.09
Praxis	Oral_Praxis	0.00	0.00	0.00
	Ideomotor apraxia	0.00	4.17	0.00
Comprehension	Token test	0.00	36.00	4.17
	Auditory comprehension nouns	0.00	10.53	0.00
	Auditory comprehension verbs	0.00	10.53	0.00
Lexico-semantics	Naming_Nouns	16.00	56.00	24.00
	Naming_Verbs	24.00	64.00	44.00
	Phonological fluency	9.09	50.00	4.55
	Pyramids and palm trees test	0.00	0.00	0.00
	Auditory lexical decisions	31.82	47.83	21.74
	Visual lexical decisions	50.00	63.64	47.83
Writing/Reading/Repetition	Word reading	4.17	20.00	8.00
	Pseudowords_Reading	0.00	20.83	4.17
	Word repetition	0.00	4.00	4.00
	Pseudoword repetition	0.00	12.50	12.00
	Writing	4.35	21.74	32.00
	Phonological discrimination	0.00	0.00	0.00

**Table 4:** Friedman test and post hoc pairwise comparisons conducted to determine whether patients’ accuracy differs before, immediately post-surgery (1 week after), and at follow-up.

Domain	Task	Friedman Test	Post Hoc Pairwise Comparisons *		
			1 w-Pre	F Up-Pre	F Up-1 w
Executive functions	Short-term memory	χ^2^(2) = 7.955, *p* < 0.05	Z = −2.75, *p* = 0.006	Z = −0.88, *p* > 0.05, n.s.	Z = −2.37, *p* = 0.019, n.s. *
	Working memory	χ^2^(2) = 8.0, *p* < 0.05	Z = −1.793, *p* > 0.05, n.s.	Z = −1.047, *p* > 0.05, n.s.	Z = −2.87, *p* = 0.007
Praxis	Oral praxis	χ^2^(2) = 2.33, *p* = 0.311. n.s.	-	-	-
	Ideomotor apraxia	χ^2^(2) = 10.242, *p* < 0.01.	Z = −2.668, *p* = 0.008	Z = −1.826, *p* > 0.05, n.s.	Z = −1.795, *p* > 0.05, n.s.
Language comprehension	Token test	χ^2^(2) = 25.683, *p* < 0.001.	Z = −4.017, *p* < 0.001	Z = −2.18, *p* = 0.029, n.s.*	Z = −3.801, *p* < 0.001
	Auditory comprehension nouns	χ^2^(2) = 7.62, *p* < 0.05	Z = −2.06, *p* = 0.036, n.s. *	Z = 0.000, *p* = 1, n.s.	Z = −2.07, *p* = 0.038, n.s. *
	Auditory comprehension verbs	χ^2^(2) = 1.077, *p* = 0.584. n.s.	-	-	-
	Phonological discrimination	χ^2^(2) = 0.368, *p* > 0.5. n.s.	-	-	-
Semantics	Noun naming	χ^2^(2) = 17.153, *p* < 0.001.	Z = −3.463, *p* < 0.001	Z = −2.415, *p* = 0.016	Z = −3.342, *p* < 0.001
	Verb naming	χ^2^(2) = 16.718, *p* < 0.001.	Z = −3.307, *p* < 0.001	Z = −2.044, *p* < 0.05	Z = −3.512, *p* < 0.001
	Fluency	χ^2^(2) = 11.919, *p* < 0.01	Z = −1.604, *p* > 0.05, n.s.	Z = −1.531, *p* > 0.05, n.s.	Z = −2.69, *p* = 0.004
	Pyramids and palm trees test	χ^2^(2) = 16.750, *p* < 0.001	Z = −2.678, *p* = 0.007	Z = −1.037, *p* > 0.05, n.s.	Z = −2.245, *p* = 0.019, n.s. *
	Auditory lexical decisions	χ^2^(2) = 7.373, *p* < 0.05	Z = −1.547, *p* > 0.05, n.s.	Z = −1.488, *p* > 0.05, n.s.	Z = −2.793, *p* = 0.005
	Visual lexical decisions	χ^2^(2) = 6.162, *p* < 0.05	Z = −0.980, *p* > 0.05, n.s.	Z = −1.510, *p* > 0.05, n.s.	Z = −2.800, *p* = 0.005
Reading/ Writing/ Repetition	Word reading	χ^2^(2) = 8.167, *p* < 0.05	Z = −1.939, *p* > 0.05, n.s.	Z = −0.405, *p* > 0.05, n.s.	Z = −2.135, *p* = 0.033, n.s. *
	Pseudoword reading	χ^2^(2) = 6.056, *p* < 0.05	Z = −2.585, *p* = 0.010, n.s. *	Z = −0.412, *p* > 0.05, n.s.	Z = −1.489, *p* > 0.05, n.s.
	Writing	χ^2^(2) = 10.79, *p* < 0.01	Z = −3.174, *p* = 0.002	Z = −2.030, *p* = 0.042, n.s. *	Z = −0.313, *p* > 0.05, n.s.
	Word repetition	χ^2^(2) = 3.5, *p* = 0.174. n.s.	-	-	-
	Pseudoword repetition	χ^2^(2) = 8.00, *p* < 0.5	Z = −1.604, *p* > 0.05, n.s.	Z = −2.410, *p* = 0.016, n.s. *	Z = −0.567, *p* > 0.05, n.s.

* Bonferroni correction for multiple comparisons (*p* = 0.0166). 1 w= 1 week post-surgery; pre = pre-surgery; F up = follow-up at 4 months after surgery.

**Table 5:** Comparison between quantitative and qualitative analysis. The two groups identified under qualitative observation as displaced/infiltrated showed statistically significant differences in terms of quantitative variables in the extraction of the left trajectories.

		Displaced Median (IQR) (n = 10)	Infiltrated Median (IQR) (n = 19)	*p*-Value
**RIGHT**	**NS**	255.5 (173–281)	290 (172–365)	0.396
	**NV**	1978 (1566–2412)	2388 (1573–2791)	0.582
	**V**	11.6 (10.6–15.4)	16.9 (11.4–18.7)	0.162
	**FA**	0.47 (0.44–0.48)	0.47 (0.46–0.48)	0.209
	**L**	136 (135–146)	141 (132–154)	0.620
	**A**	3.51 (3.4–3.58)	3.35 (3.23–3.57)	0.089
	**AD**	1.25 (1.24–1.32)	1.25 (1.19–1.27)	0.223
	**RD**	0.57 (0.55–0.6)	0.54 (0.52–0.56)	0.116
	**MD**	0.8 (0.75–0.83)	0.76 (0.75–0.81)	0.356
**LEFT**	**NS**	198 (159–296)	35 (2–96)	**<0.001**
	**NV**	1956 (1760–2453)	551 (83–917)	**<0.001**
	**V**	13 (10.3–14.9)	3.6 (0.6–6.6)	**<0.001**
	**FA**	0.45 (0.43–0.49)	0.40 (0.36–0.44)	**0.004**
	**L**	137 (135–145)	105 (86–126)	**<0.001**
	**A**	3.49 (3.27–3.79)	3.89 (3.27–4.37)	0.324
	**AD**	1.31 (1.28–132)	1.29 (1.24–1.45)	0.782
	**RD**	0.58 (0.54–0.6)	0.68 (0.59–0.71)	**0.024**
	**MD**	0.82 (0.8–0.84)	0.85 (0.79–0.96)	0.200

NS: number of streamlines; NV: number of voxels; V: fascicle volume; FA: fractional anisotropy, L: length of streamlines; A: angle of streamlines; AD: axial diffusivity; RD: radial diffusivity; MD: medial diffusivity.

**Table 6:** Association between neuropsychological altered performance and microstructure analysis at immediate post-surgery and follow-up assessments.

	Immediately Post-Surgery		Follow-Up	
	LH IFOF	RH IFOF	LH IFOF	RH IFOF
Naming verbs	NV, ρ = 0.541, *p*-value = 0.005 NS, ρ = 0.501, *p*-value = 0.011 MD, ρ = 0.414, *p*-value = 0.049	AD, ρ = 0.631, *p*-value < 0.001 MD, ρ = 0.455, *p*-value = 0.022	NV, ρ = 0.622, *p*-value = 0.001 NS, ρ = 0.564, *p*-value = 0.003 L, ρ = 0.47, *p*-value = 0.017 AD, ρ = 0.440, *p*-value = 0.036	AD, ρ = 0.569, *p*-value = 0.003 MD, ρ = 0.435, *p*-value = 0.030
Token test	NV, ρ = 0.426, *p*-value = 0.034	AD, ρ = 0.445, *p*-value = 0.026		FA, ρ = 0.413, *p*-value = 0.045
Naming nouns	-	MD, ρ = 0.651, *p*-value < 0.001 AD, ρ = 0.494, *p*-value = 0.012	NV, ρ = 0.466, *p*-value = 0.019	MD, ρ = 0.611, *p*-value = 0.001
P&P		A, ρ = −0.461, *p*-value = 0.047		
STM	L, ρ = 0.492, *p*-value = 0.014	AD, and ρ = 0.518, *p*-value = 0.010		
Writing	-	AD, ρ = 0.457, *p*-value = 0.032 MD, ρ =- 0.438, *p*-value = 0.042		
IMA	-	MD, ρ = 0.455, *p*-value = 0.033 NS, ρ = −0.428, *p*-value = 0.037		
Phonological fluency			L, ρ = 0.574, *p*-value = 0.008	
Lexical decisions				FA, ρ = 0.444, *p*-value = 0.039

NS: number of streamlines; NV: number of voxels; V: fascicle volume; FA: fractional anisotropy, L: length of streamlines; A: angle of streamlines; AD: axial diffusivity; RD: radial diffusivity; MD: medial diffusivity.

## Data Availability

The datasets generated during and/or analyzed during the current study are available from the corresponding author on reasonable request.
